# *In vitro* evaluation of anti-angiogenesis property of anti-VEGFR2 nanobody-conjugated H40-PEG carrier loaded with methotrexate

**DOI:** 10.22038/IJBMS.2022.67038.14701

**Published:** 2022-12

**Authors:** Seyed Masih Adyani, Hamid Rashidzadeh, Mahdi Behdani, Seyed Jamal Tabatabaei Rezaei, Ali Ramazani

**Affiliations:** 1 Pharmaceutical Biomaterials Department, School of Pharmacy, Zanjan University of Medical Sciences, Zanjan, Iran; 2 Department of Molecular Medicine, Pasture Institute of Iran, Tehran, Iran; 3 Department of Chemistry, University of Zanjan, Zanjan, Iran; 4 Cancer Gene Therapy Research Center, Zanjan University of Medical Sciences, Zanjan, Iran; 5 Department of Pharmaceutical Biotechnology, Zanjan University of Medical Sciences, Zanjan, Iran

**Keywords:** Angiogenesis inhibitors, Methotrexate, Polymer, Single-domain antibodies, Vascular endothelial - Growth factor receptor-2

## Abstract

**Objective(s):**

In this study, Boltorn® H40-PEG-MTX-anti-VEGFR2 nanobody was fabricated in which nanobody was selected for blocking the receptor, H40 as a nanocarrier for delivery of methotrexate (MTX) to the tumor cells, and polyethylene glycol (PEG) moieties for improving the blood circulation time and safety.

**Materials and Methods:**

The synthesis process of the nanosystem has been characterized by different analytical methods.

**Results:**

The prepared nanoplatform exhibited high drug loading capacity, excellent colloidal stability, and an average particle size of around 105 nm. MTX was successfully conjugated through ester bonds and its release profile clearly showed that the ester bond is in favor of releasing the drug in acidic pH (5.5). The cytotoxicity of the developed nanoplatform exhibited great anti-cancer activity against MCF7 and KDR293 (cells with overexpressed anti-VEGFR2 NB receptors) cell lines while no deleterious toxicity was observed for nanocarrier against HEK293 normal cells. Furthermore, both hemolysis and LD_50_ assay results confirmed the hemocompatibility and biocompatibility of the developed nanoplatform.

**Conclusion:**

The most striking result to derive from the data is that the designed nanoplatform could potentially inhibit cell migration and invasion and the anti-angiogenesis properties of the developed nanoplatform may serve as a promising nanosystem to suppress the formation of blood vessels around tumor cells and consequently inhibit tumor progression.

## Introduction

Angiogenesis is a complex and intricate regulatory process and is defined as the formation of new blood vessels from the existing vasculature. This process involves the migration, growth, and differentiation of endothelial cells located within the walls of blood vessels and is a fundamental phenomenon for the growth and survival of solid neoplasms. The process of angiogenesis is controlled by chemical signals in the body and can stimulate the formation of new blood vessels (1). Tumor angiogenesis is the proliferation of blood vessels to supply nutrients and oxygenation for tumor growth (2). Angiogenesis is essential not only for the growth of persistent tumors but also for metastasis. Since an adequate vascular response is critical for the early development and continued growth of solid tumors, it is essential to pay attention to the use of angiogenesis process inhibition in addition to the current treatments to prevent the progression of malignant neoplasm (3). Angiogenesis inhibitors prevent the formation of blood vessels. Typically, the excitatory and inhibitory effects of these chemical signals are balanced so blood vessels are formed when needed (4).

In angiogenesis migration, growth, and differentiation of endothelial cells, the usual mechanism of this process, is controlled by chemical signaling pathways (5). In the cell signaling pathway, vascular endothelial growth factor (VEGF) is critical for vascular development. The VEGF family consists of five members (VEGF-A, VEGF-B, VEGF-C, VEGF-D, and platelet growth factor (PLGF)). These factors show different affinities for VEGF receptors (VEGFR-1, VEGFR-2, and VEGFR-3( (6, 7).

Anti-angiogenesis drugs, especially anti-vascular endothelial growth factor (VEGF) agents on the market, are associated with unexpected toxicities. The prevalence and severity of these toxicities have many variables in different studies. Among them, bleeding is one of the most severe. Bevacizumab has the highest incidence of bleeding complications, especially epistaxis and gastrointestinal bleeding. Although the incidence of severe bleeding has not been consistently established during treatment with Bevacizumab, light bleeding has occurred in the experimental part of most trials. Cases of severe pulmonary hemorrhage have been reported in patients with lung cancer. These events occurred mainly within the tumor and were significantly related to squamous cell histology (8). Experiments with smaller inhibitors of tyrosine kinase molecules, such as Sunitinib or Sorafenib, showed an overall reduction in blood vessel counts, but in many cases were significantly higher than controls (9).

To date, the development of effective treatments for cancers remains a challenge. Conventional chemotherapy and radiation therapy have a powerful effect on killing tumor cells, but in their way, they also kill healthy cells. Therefore, extensive attention has been paid to the development of more effective treatment options with a targeted approach (10-12). Over the years, antibodies have been used as antiviral therapy and later as valuable research and clinical diagnostic tools. The first injection of monoclonal antibodies (mAbs) into patients dates back about 30 years ago (13). But mAbs from mice or rats cause immunogenic problems in patients. MAb-based treatment has achieved considerable success in the treatment of many cancers. These mAbs are selected based on their ability to disrupt the normal function of their targets in tumor cells. Intact mAbs, containing a fully functional Fc domain can induce antibody-dependent cell cytotoxicity (ADCC). In addition, mAbs have the potential for the detection of tumors through molecular imaging (14).

The mechanism of bleeding due to anti-VEGF agents is complex and has not yet been fully elucidated. The tendency to bleeding after inhibition of VEGF-dependent signal opens up the physiological activity of VEGF on the vascular walls and possibly on the clotting system. The hypothesis is that inhibition of VEGF reduces the capacity of endothelial cells to regenerate after a stroke (15). Therefore, attention to receptor blocking in angiogenesis (VEGFR2) instead of its ligand was on the agenda. Although currently, a monoclonal anti-VEGFR2 antibody is on the market, studies on antibodies have focused on reducing the size of these peptides to address the problem of bioavailability and limited tumor distribution, which eventually led to the discovery of a new type of antibody in camels that is naturally light-chain; they do not have their first constant domain (CH1) in the heavy chain (16). This VHH molecule with a size of less than 15 kDa and dimensions in the nanometer range (~ 2.5 nm in diameter and ~ 4 nm in height) was named nanobody (NB). Concerning the properties of nanobodies, an alternative to the current antibodies is found as a targeted agent of intelligent drug delivery systems.

These heavy-chain-only antibodies (HcAbs) are involved in antigen detection compared with conventional antibodies, despite having only a single variable domain (VHH) (17). VHH has been shown to retain antigen-binding potential and is considered the smallest part of natural antigen binding (18, 19). Except for the dimensions in the single-digit nanometer range of nanobodies, we can mention their unique properties such as high stability and solubility, specificity and affinity, and excellent properties against all possible targets, including tumor markers.

From the outset, the potential of NBs has been evaluated as therapeutic agents for cancers, by NB targeting cell surface targets to inactivate the signaling cascades. Therapeutic agents and different formats of NB-based drug delivery systems are as follows: NBs with intrinsic therapeutic activities (20, 21), nanobody conjugated with toxins (22), nanobody conjugated with liposomes (23), polymer nanoparticles (dendrimers, nanospheres, nanocapsules, and nanoparticles conjugated with albumin nanoparticles) (24, 25) and conductive nanobody with carrier (26). Among the drug delivery systems, one of these widely used systems is nanobody conjugation with polymer nanoparticles.

The drug delivery system using nanoparticles (e.g., polymer nanoparticles) has paid serious attention to drug delivery by targeting cell surface ligands (26-29). These systems affect tumor cells using active and inactive mechanisms with enhanced permeability and retention (EPR) (30-32). As one of the most promising nanoparticle systems, polymeric dendrimers were used for drug delivery systems due to their ability to combine drugs in nanosystems, improve bioavailability, solubility, and drug retention time, and also overcome the issue of multiple drug resistance (MDR). However, this system has some inherent limitations, including poor targeting efficacy. To overcome these limitations, the introduction of various target ligands or antibodies and nanobodies in the drug delivery system makes it possible to deliver the drug inside or on the surface of tumor cells through the endocytosis receptor (33, 34).

For most effective drugs in animal experiments, unknown and potentially adverse side effects impede clinical use. Therefore, the effects of methotrexate (MTX), an immunosuppressant drug, will be investigated on angiogenesis. MTX has been used in cancers for a long time, and its side effects are well known. MTX is a chemotherapeutic agent introduced in 1958 to treat leukemia. It has been used as a folic acid antagonist, an anti-cancer drug, and for systemic rheumatic diseases (35, 36).

For cancer, MTX competitively inhibits the dihydrofolate reductase (DHFR) enzyme, which participates in the synthesis of tetrahydrofolate (37). The affinity of MTX for DHFR is about 1000 times more than folate. Folic acid is essential for the synthesis of DNA. MTX, therefore, inhibits the synthesis of DNA, RNA, thymidylates, and proteins (36). 

Due to the recent issue of using nanobodies for active targeting of carriers and the lack of extensive literature sources on this type of carrier, the focus of this study is on the conjugation of nanobodies to polymer dendrimer (H40-PEG) carrier targeting VEGFR2 on the cell surface. VEGFR2 nanobodies are ligands with a high affinity to VEGFR2, one of the ligands in angiogenesis.

The usage of anti-VEGFR2 NB with Boltorn H40 and dual delivery of NB and MTX as a folic acid analog were not reported in the literature. Therefore, in this study, we aimed to investigate the targeted release of MTX and inhibit angiogenesis using an anti-VEGFR2 nanoparticle targeting agent in the H40-PEG system. 

## Materials and Methods

Boltorn H40, Poly ethylene Glycol 2000 (PEG 2000), N-(3-Dimethylaminopropyl)-N0-ethyl-Carbodiimide hydrochloride (EDC) and N-Hydroxysuccinimide (NHS), 3-(4, 5- dimethylthiazol-2-yl)-2, 5-diphenyl tetrazolium bromide (MTT) and 4-(dimethylamino) pyridine (DMAP) were purchased from Sigma Aldrich Chemicals, (St. Louis, MO, USA). Methotrexate was provided by Alvand Pharmaceutical Nanotechnologists Company, and Anti-VEGFR2 nanobody was cloned and purified at Pasteur Institute of Iran. All other solvents were purchased from Emertat Chimi Company (Tehran, Iran).

S***ynthesis of H40-succinic anhydride***

In brief, a certain amount of succinic anhydride and H40 was added to 20 ml of chloroform (CF) and tetrahydrofuran (THF) mixture with a ratio of 1 to 2. After that, EDC/NHS (165/100 mg) was added to the mixture and stirred at room temperature for 24 hr. The crude product was then obtained using diethyl ether and dried by vacuum oven at 50 ºC.

***Synthesis of H40-succinic anhydride-PEG***

For conjugation of PEG to H40-COOH, a certain amount of the product of the previous step was mixed with 38.24 mg EDC and 23 mg NHS in 50 ml DMSO solvent to activate the carboxylic acid group, and then after stirring for 6 hr at room temperature, 10 mg PEG was added to the reaction medium and stirred for 24 hr. Then it was dialyzed against deionized water to remove impurities and unreacted materials from the product. Finally, the solution inside the dialysis bag was pulverized using a freezer. 

***Conjugation of H40- succinic anhydride-PEG to MTX***

The previously mentioned procedure was used with minor changes, except that instead of activation of the carboxylic acid groups of H40-succinic anhydride, carboxylic acid groups of MTX were activated. In brief, 10 mg of MTX was mixed with 50 ml solution containing EDC/NHS (1:2 molar ratio) and stirred for 6 hr at room temperature in the dark. Then the prepared crude materials obtained from the previous step were added to the reaction mixture and stirred for 24 hr under the same reaction conditions (37 °C and dark). Finally, the mixture was dialyzed against distilled water for 48 hr to obtain the impurity-free H40-succinic anhydride-PEG-MTX.

***Conjugation of NB to H40-succinic anhydride-PEG-MTX***

**Figure 1:**
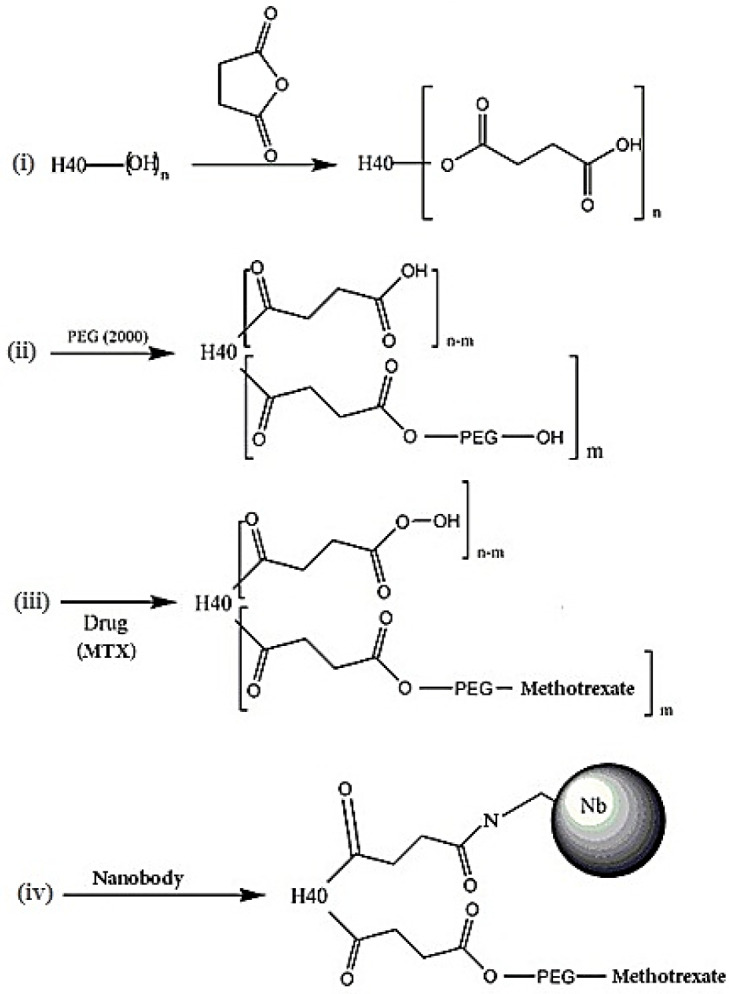
Schematic representation of synthesis route of H40-PEG-MTX-NB and corresponding steps. (i) Synthesis of H40-Succinic Anhydride; (ii) Synthesis of H40- Succinic Anhydride-PEG; (iii) Synthesis of H40- Succinic Anhydride-PEG-MTX and (iv) Synthesis of H40- Succinic Anhydride-PEG-MTX-NB

MTX: Methotrexate; PEG: Poly ethylene Glycol; NB: Nanobody

For conjugation of NB, the above steps were repeated. Briefly, a certain amount of the H40-succinic anhydride-PEG-MTX was used with EDC / NHS in DMSO solvent to activate the remaining carboxylic acid group of H40-succinic anhydride, and then after stirring for 6 hr at room temperature, NB was added to the reaction medium and stirred for 48 hr to ensure that the conjugation reaction processed admirable. Then the reaction solution was placed in a dialysis bag to remove impurities and unreacted materials from the product. The synthesis and conjugation process schematically has been shown (Figure 1). The final conjugates were separated from unbound NB by gel permeation chromatography. Gel permeation chromatography using a Superdex G75 column was performed. The conjugates were eluted at 1 ml/min using a 50 mM phosphate/150 mM NaCl buffer, and the eluted fractions were monitored at 280 nm. Finally, the solution inside the dialysis bag was pulverized using a freezer for characterization and other *in vitro* assays.

***Characterization of the PEG–H40 and MTX conjugated PEG–H40 dendrimer***

The structures of the copolymer and conjugate were determined using proton nuclear magnetic resonance spectroscopy (^1^H NMR) in CDCl3 at 400 MHz (Bruker, Avance 400), Fourier Transforms Infrared Spectroscopy (FT-IR) (Bruker, Tensor 27), and UV-Vis spectrophotometer.

***Determination of particle size and stability***

The particle size distribution of the prepared micelles was determined by Dynamic Light Scattering (DLS) using a nano/zeta sizer (Malvern Instruments, Nano ZS, Worcestershire, UK).

To determine the stability of the carrier containing the drug, DLS is used to measure the particle size at different intervals for stability. The stability of the nanobody structure after conjugation was analyzed using SDS-PAGE.***e ***

The amount of drug loading on the prepared carrier is determined with an ultraviolet (UV-Vis) spectrometer. To determine the release rate of the drug at different pH values (pH of 5.5 and 7.4). Briefly, 5 mg of H40-PEG-MTX were dispersed in 1 ml PBS and the resulting suspension was placed within a dialysis bag (Mw 12 kDa) and incubated at 37 °C. Then, at predetermined time intervals, 2 ml of the dialysate was taken out and replaced by 2 ml of fresh PBS. The concentration of MTX in the dialysate was measured at 304 nm using a UV-Vis spectrophotometer. 

***Cytotoxicity assay***

*LD*_50_

BALB/c mice (9–10 weeks old) were used for this test. Firstly, *in vivo* survival assay was used to examine the biosafety of H40-PEG according to previously established research (38). In detail, H40-PEG in concentration of 125 mg/kg, 250 mg/kg, 500 mg/ kg, and 1000 mg/kg were injected via tail vain to BALB/c mice. The Kaplan−Meier curve of the survival assay of H40-PEG will be depicted to show the survival percentage. All experimental protocols were approved by the institutional Ethics Committee of Zanjan University of Medical Sciences (Ethical Code IR.ZUMS.REC.1397.321).

*MTT assay*

MTT assay is used to evaluate cell survival after treatment with an external agent. This technique is based on the reduction of MTT yellow substrate to purple deposition of formazan by mitochondrial reductase enzymes in living cells. The intensity of the dye produced after dissolving formazan crystal in organic solvents such as dimethyl sulfoxide can be measured by spectrophotometry and has a direct relationship with the number of living cells.

Cell count was performed using a Neubauer slide and 50,000 cells were cultured in 96-well plates with a final volume of 100 μl of complete dedicated culture medium. Each concentration is tested in triplicate. Then 20 μl of MTT was added with a concentration of 5 mg/ml (dissolved in PBS buffer) to each well. In an incubator at 37 °C with 5% carbon dioxide for 4 hr and then draining the wells and adding 100 μl of DMSO to each well and measuring cell uptake at 570 nm, the percentage of cell survival using the ratio adsorption in the cells of the treatment group is calculated to the control group.

*Red blood cell destruction assay*

A hemolysis test was performed for evaluating the biocompatibility of the copolymer. In summary, 1 ml of human red blood cells is obtained by separating serum from human blood by centrifugation and washing three times with sterile PBS solution. It is then diluted with PBS, then mixed with 0.5 ml of a solution containing the sample. The concentration of the samples should be 10 mg/ml. Sodium lauryl sulfate (SLS) is used as the positive control and PBS solution as the negative control. The samples are shaken in an incubator shaker at 37 °C for 4 hr. Finally, the samples are centrifuged at 2700 rpm for 5 min and the supernatant is removed and its absorption was read at 540 nm. 

***Anti-angiogenesis assay***

*Immigration assay*

The transit migration test is used to determine the effects of NB on the movement and delay of cell migration power (39). HUVEC Cells (50,000/well) are located on the upper surface of the transvolume sheet. Then 500 μl of DMEM with different concentrations of NB (ng/ml) was added to the bottom layer and 500 μl of DMEM was added to the top layer. PBS and an irrelevant NB were used as negative controls. After 16 hr, the bottom surface was washed and stained with Giemsa. The cells were counted in five randomly-chosen fields via light microscopy.

*Invasion assay*

For an invasion assay, a thin layer of collagen I was applied to the top surface of the wells. KDR cells (5×10^4^ cells/well) were plated on collagen layers. 500 microliters of DMEM with 400, 800, and 1600 ng/ml of NB were added to the bottom layer of the well, and 500 microliters of DMEM were added to the upper layer. PBS and an indirect NB were used as negative controls. After about 12 hr, the collagen was removed from the upper surface with a sterile swab and the cells at the lower surface were fixed with methanol and stained with Giemsa. The cells were counted in five randomly-chosen fields via a microscope (39).

***Statistical analysis***

All the data were expressed as mean ± standard deviation (SD). A one-way ANOVA test was employed for statistical analysis using SPSS software version 16 and *P*<0.05 was considered statistically significant.

## Results

***Synthesis and Characterization of H40-MTX***

**Figure 2:**
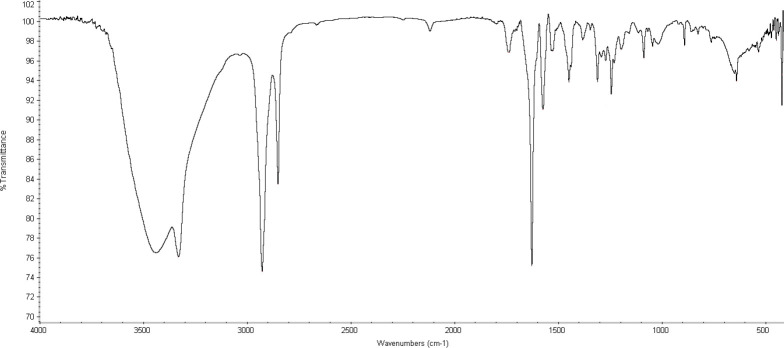
FT-IR spectra of H40-PEG-MTX

FT-IR: Fourier transforms infrared spectroscopy; PEG: Poly ethylene Glycol; MTX: Methotrexate

Figure 2 shows the FT-IR spectra of H40-PEG-MTX-NB. It clearly shows that the clear characteristic bands at 1628 cm^-1^ show the conjugation of H40 with NB through am amide bond formation (40). A distinct peak at 1088 cm^-1^ is attributed to the C-O stretching bond and peaks at 2851 cm^-1^ and 2927 cm^-1^ are corresponding to the methyl and methylene groups of PEG which represented the successful conjugation of PEG. Additionally, PEG, as well as MTX conjugation, occurred through an ester bond formation. Accordingly, the peak at 1740 cm^-1^ can be attributed to the formation of ester bonds through the synthesis of H40-PEG and H40-PEG-MTX (41). Moreover, the bonds at around 1448–1574 cm^-1^ are assigned to the stretching vibrations of aromatic rings of MTX molecules. Of note, N–H stretching vibrations of amine groups was also observed at 3330 cm^-1^ (36) While the distinct peaks at 1045/3438 cm^-1^ is corresponding to the hydroxyl of H40 macromolecules. Taken together, FTIR results indicate that the conjugation of PEG, MTX, and NB successfully occurred to form H40-PEG-MTX-NB nanoparticles.  

**Figure 3:**
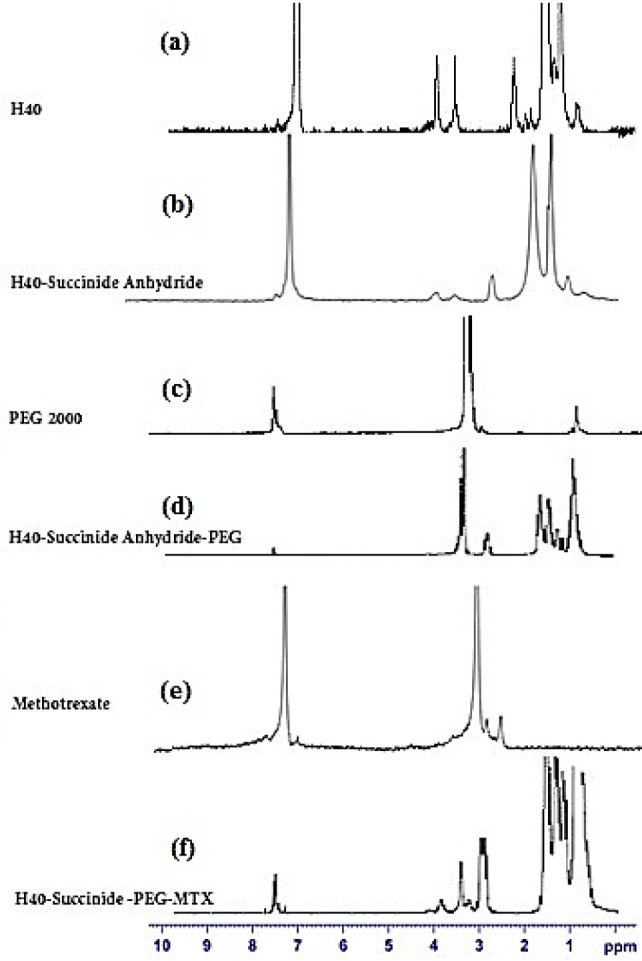
^1^H NMR spectra of H40, PEG, MTX, H40-succinide anhydride, H40-succinide anhydride-PEG, and H40-succinide anhydride-PEG-MTX

HNMR: Proton nuclear magnetic resonance; PEG: Poly ethylene Glycol; MTX: Methotrexate

The HNMR spectra of H40 and conjugated moieties are presented in Figure 3. It is clear that Boltorn® H40 had two characteristic peaks at 1.20-125 ppm and 4.23 ppm and corresponded to the protons of the methyl and methylene groups, respectively (Figure 3a) (42). Succinic anhydride conjugated to Boltorn® H40, and peaks at 2.45 ppm are attributed to the methylene groups of succinic anhydride and underline the successful conjugation process (Figure 3b) (43). Then, PEG moieties were added to the H40-succinic anhydride to form H40-PEG, Figure 3c represented the HNMR spectra of PEG in which a peak at 3.61 ppm is the representative peak of PEG molecules. Accordingly, H40-succinic-PEG is fabricated in which Figure 3d indicated the characteristic peak of PEG (3.65 ppm) and highlighted that PEG was conjugated to H40-succinic anhydride. MTX acts as a dual function, chemotherapeutic and targeting role. As shown in Figure 3d, peaks between 2 ppm to 4 ppm along with 7–8 ppm are the main peaks of MTX (40). Finally, MTX was decorated onto H40-succinic-PEG to create the H40-succinic-PEG-MTX and its HNMR spectrum has been displayed in Figure 3f. All characteristic peaks of MTX (6–8 ppm) are also observed in H40-succinic-PEG-MTX and confirmed that MTX has been bonded to the H40-succinic-PEG (40). Altogether, HNMR results underline that the conjugation of PEG and MTX successfully occurred to form H40-PEG-MTX nanoparticles. 

***Characterization of the nanobody conjugated PEG–H40-MTX dendrimer***

**Figure 4:**
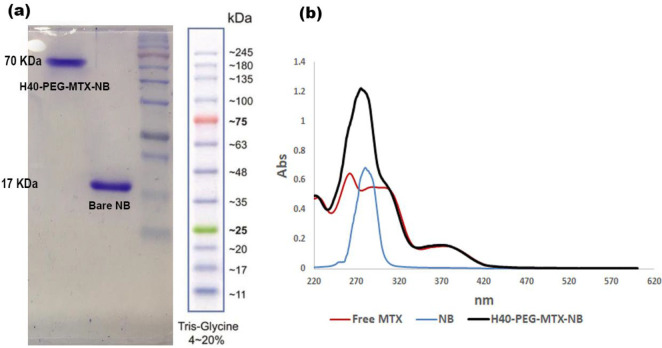
(a) SDS-PAGE gel stained with coomassie blue of Bare NB and H40-PEG-MTX-NB. (b) UV spectrum of MTX, NB, and H40-PEG-MTX-NB

SDS-PAGE: Sodium dodecyl-sulfate polyacrylamide gel electrophoresis; NB: Nanobody; PEG: Poly ethylene Glycol; MTX: Methotrexate

For verifying that NB has been conjugated to the fabricated carrier, we used UV spectrum and SDS-PAGE. Figure 4a clearly shows that anti-VEGFR2 NB has a molecular weight of around 17 KDa and by its conjugation to the fabricated nanocarrier, molecular weight shifted to around 70 KDa. UV spectra of MTX, NB, H40-PEG-MTX-NB, and H40-PEG are depicted in Figure 4b. MTX exhibited two characteristic peaks at around 371 nm and 259 nm. NB exhibited a distinct peak at around 281 nm. There is a minor shift in the wavelength of H40-PEG-MTX-NB concerning both MTX and NB. This could be another verification for NB and MTX conjugation to the H40-PEG carrier (36). In addition, GPC results showed that the average molecular weight and polydispersity index of the conjugated polymer was 69.34 kDa and 1.3 kDa, respectively.

***Determination of particle size and stability***

**Figure 5:**
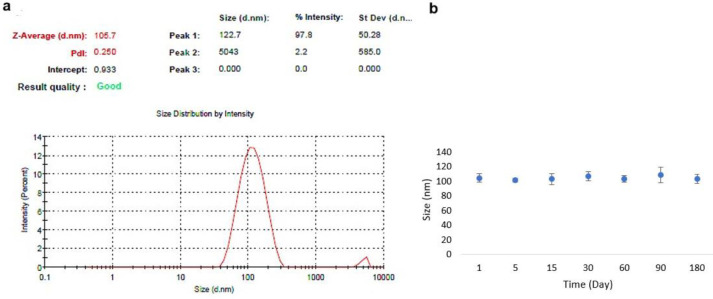
(a) Hydrodynamic size distribution of H40-PEG-MTX-NB and (b) Size monitoring of H40-PEG-MTX-NB up to 180 days

**Figure 6:**
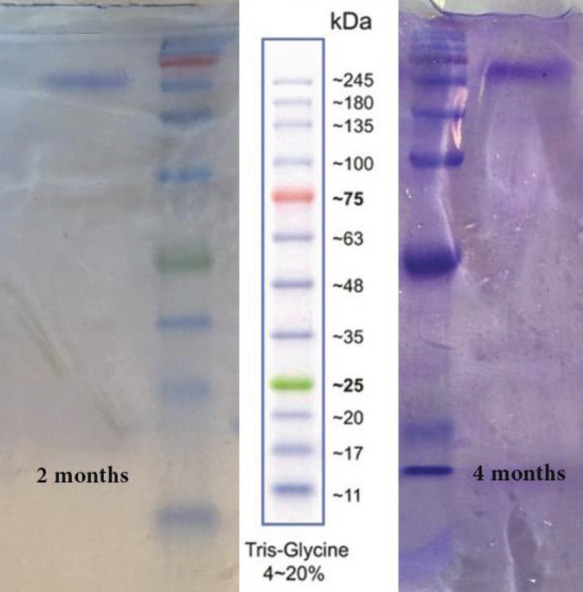
SDS PAGE gel of H40-PEG-MTX-NB after 2 and 4 months

SDS-PAGE: Sodium dodecyl-sulfate polyacrylamide gel electrophoresis; NB: Nanobody; PEG: Poly ethylene Glycol; MTX: Methotrexate

It has been well known that the hydrodynamic diameter of the developed nanomaterials has a great impact on their biological application, as their interactions with cells along with their cellular uptake efficiency, and internalization are strongly affected by the hydrodynamic diameter of nanoparticles. Accordingly, the hydrodynamic diameter of H40-PEG-MTX-NB nanoplatforms has been shown in Figure 5a. The prepared nanoparticles show a size of about 105.7 nm with a polydispersity index (PDI) of 0.250 which is suitable for biological applications. Also, the stability of synthesized H40-PEG-MTX-NB was monitored for up to 180 days by DLS (Figure 5b). In Figure 6, the SDS-PAGE analysis of the H40-PEG-MTX-NB after 2 and 4 months of conjugation has been shown.

***Drug loading and release profile***

**Figure 7:**
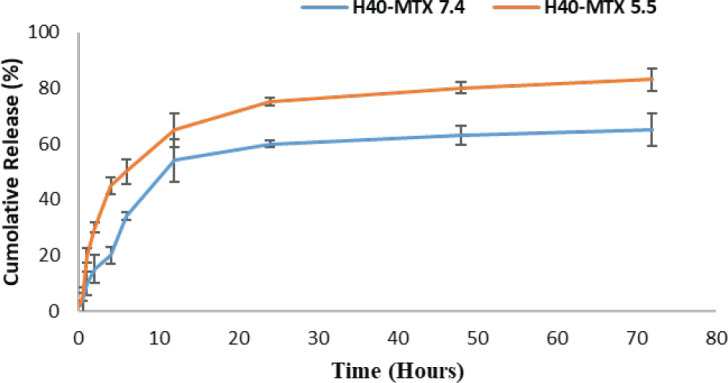
Release profile of H40-PEG-MTX-NB in neutral (pH = 7.4) and acidic PBS solutions (pH = 5.5) (*P*<0.01)

NB: Nanobody; PEG: Poly ethylene Glycol; MTX: Methotrexate; PBS: Phosphate-buffered saline

According to the obtained data, the drug loading was 8%. Also, in this work, the effect of pH on the release behavior of drug-conjugated H40-PEG was examined. The release behavior of the MTX -conjugated H40-PEG was studied in neutral pH (pH 7.4) and acidic PBS solutions (pH 5.5). Figure 7 shows the release profiles of MTX from the drug–conjugated copolymer, at pH 7.4 and 5.5. As inferred from Figure 7, around 85% (± 4) is the cumulative release in pH 5.5 in difference to 65% (± 6.5). There is a significant difference between the release profile of MTX from H40-PEG in pH 7.4 and 5.5 (*P*<0.01). 

***Cytotoxicity assay***

*LD*_50_

**Figure 8:**
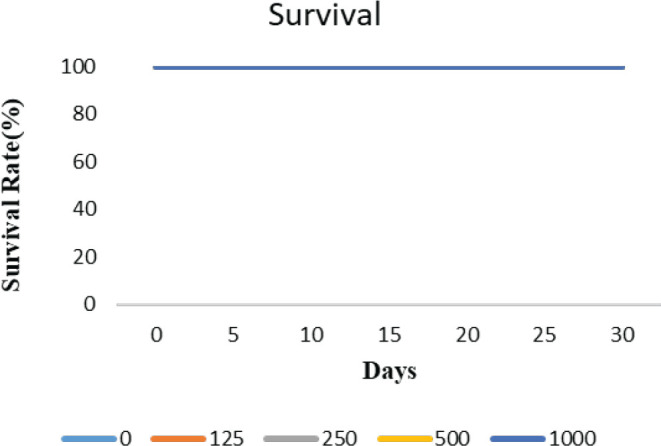
Kaplan−Meier curve of survival assay of H40-PEG

BALB/c mice (9–10 weeks old) were used for this assay. Firstly, the *in vivo* survival assay was used to examine the biosafety of H40-PEG. In detail, H40-PEG in concentrations of 125 mg/kg, 250 mg/kg, 500 mg/ kg, and 1000 mg/kg was injected via tail vain to BALB/c mice. Next, to determine the LD_50_ in each dose, four mice were injected. The mortality, body weight, and behavior of mice were monitored for up to 30 days. Figure 8 shows the Kaplan−Meier curve of the survival assay result of H40-PEG which indicated that all mice survived and no mortality was observed. This assay highlighted the biosafety of fabricated nanoparticles for further clinical investigation. 

*MTT assay*

**Figure 9:**
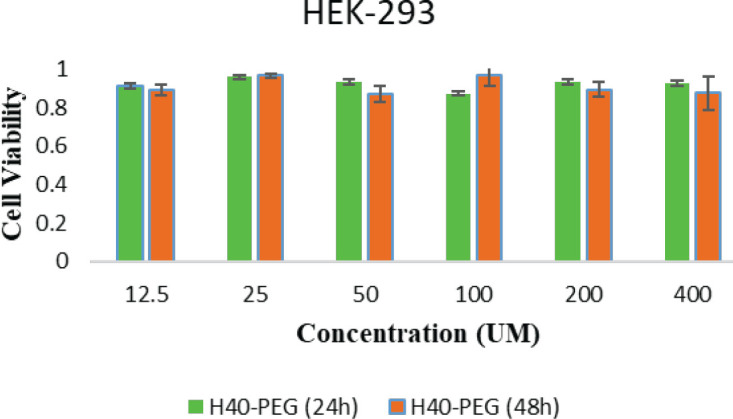
Cell Survival of H40-PEG in HEK-293 cell line (*P*>0.1)

HEK: Human embryonic kidney cells; PEG: Poly ethylene Glycol

**Figure 10:**
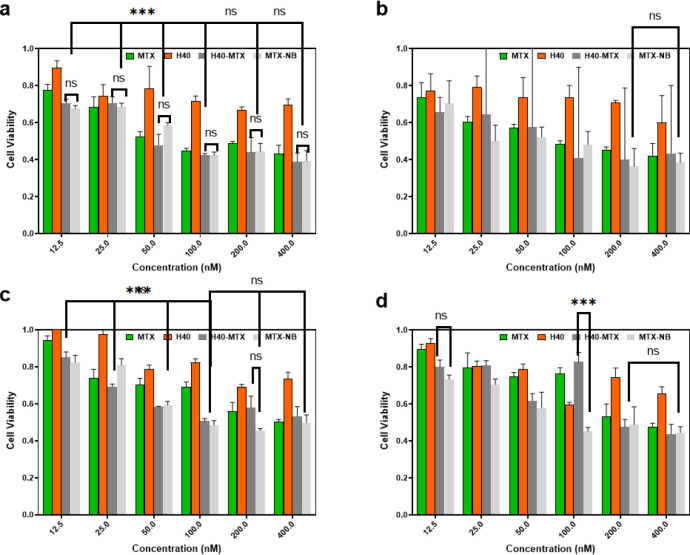
Cell Survival of MTX, H40-PEG, H40-PEG-MTX, and H40-PEG-MTX-NB at concentrations of 12.5, 25, 50, 100, 200, and 400 nM in 5% CO_2_ in (a) MCF7 cell line after 24 hr, (b)MCF7 cell line after 48 hr, (c) KDR cell line after 24 hr, (d) KDR cell line after 48 hr. Increased toxicity of the H40-PEG-MTX and H40-PEG-MTX-NB with increased concentrations is evident. The cytotoxicity rate of H40-PEG was negligible against cancer cells (in either MCF7 or KBR cell lines). For simplicity, significance bars are summarized and 48 hr graphs are similar to 24 hr for each cell line (*** = *P*<0.001, ** = *P*<0.01, ns= *P*-value>0.1)

NB: Nanobody; PEG: Poly ethylene Glycol; MTX: Methotrexate

For drug delivery and other biomedical uses, toxicity is a critical aspect to consider when evaluating their applications. For biomedical applications, dendrimeric polymers are intentionally engineered to interact with cells, and it is essential to ensure that these engineering nanomaterials do not have any adverse effects. The cytotoxicity potential of MTX, H40-PEG, H40-PEG-MTX, and H40-PEG-MTX-NB was performed against HEK-293 (human embryonic kidney 293 cells), MCF7 (human breast cancer cell), and KDR293 (overexpressed for VEGFR2 receptors) cell line over a wide range of concentrations. The cells were incubated for 24 and 48 hr at concentrations of 12.5–400 nM in 5% CO2. Figure 9 shows the cell viability results of H40-PEG against the HEK-293 cell line. The result clearly shows that the drug carrier is safe and has no toxicity against HEK-293 cells. Also, there were no differences in MTT results at different concentrations of tested nanoparticles in which cell viability was close to almost 100% (*P*-value>0.1). This means that PEG moieties could mask the possible toxicity of nanocarrier and improve its biocompatibility. Similarly, Jafari Iri Sofla *et al*. developed PEG-grafted polyamidoamine dendrimer (PAMAM) decorated with HER2-targeting nanobody, and the result disclosed that PEGylation decreased the cytotoxicity of dendrimers (44). By improving the biosafety of the developed nanocarrier, its potential cytotoxicity against cancer cells was also investigated. Accordingly, Figure 10 shows and compares the cell viability of MTX, H40-PEG, H40-PEG-MTX, and H40-PEG-MTX-NB. The toxicity of the H40-PEG-MTX and H40-PEG-MTX-NB increased at high concentrations. H40-PEG-MTX-NB exhibited concentration-dependent cytotoxicity while the cytotoxicity rate of H40-PEG was negligible against cancer cells. There were no significant differences between H40-PEG-MTX and H40-PEG-MTX-NB in either MCF7 or KBR cell lines (*P*-value>0.1). 

*Red blood cell destruction assay*

**Figure 11:**
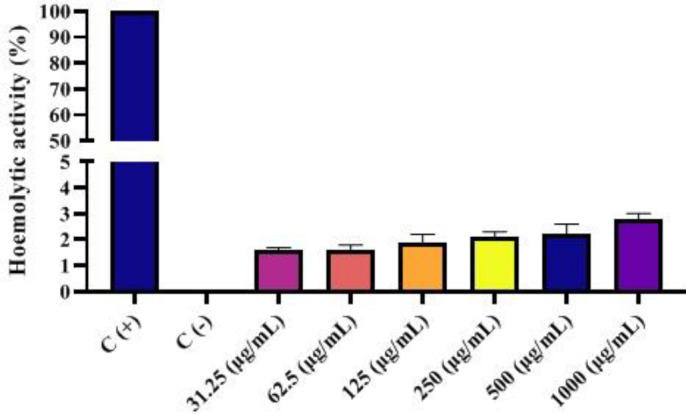
Hemolysis Assay of H40-PEG

Hemolysis assay was considered a useful, simple, and rapid technique to estimate the hemocompatibility of any developed nanoplatforms. As expected, the negligible effect of hemolytic activity is considered for the developed nanocarrier over the broad range of concentrations. The hemolytic activity of the specimens was further quantitatively specified by evaluating the supernatant absorbance at 540 nm (hemoglobin). The hemolysis potential of H40-PEG at different concentrations has been shown in Figure 11. 

***Anti-angiogenesis assay***

*Immigration assay*

**Figure 12:**
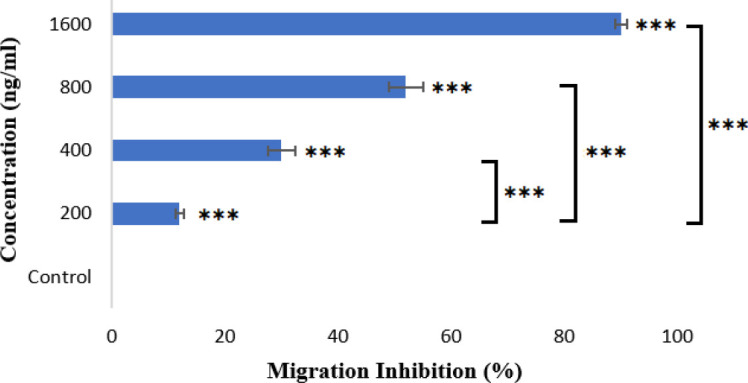
Effect of various concentrations of NB conjugated copolymer in preventing HUVEC cell migration in transwells measured against control cells. (*** = *P*<0.001), EC50 = 58.5 nM

As shown in Figure 12, different concentrations of NB conjugated copolymer, ranging from 200 to 1600 ng/ml, could significantly inhibit the motility of HUVEC cells compared with the control group of cells treated with a similar concentration of an irrelevant NB. The NB-conjugated copolymer could inhibit the migration of HUVECs but the degree of inhibition was dependent on the concentration of developed H40-PEG-MTX-NB nanoplatforms (*P*<0.001, EC_50_ = 58.5 nM). In other words, by increasing the concentration of H40-PEG-MTX-NB nanoplatform, the percentage of (%) migration inhibition of HUVECs increased as well. The H40-PEG-MTX-NB nanoplatform with 1600 ng/ml is found to have presented the highest level of inhibition which underlines the usefulness of developed nanoplatforms as efficient anti-angiogenic agents for cancer treatment.

*Invasion assay*

**Figure 13:**
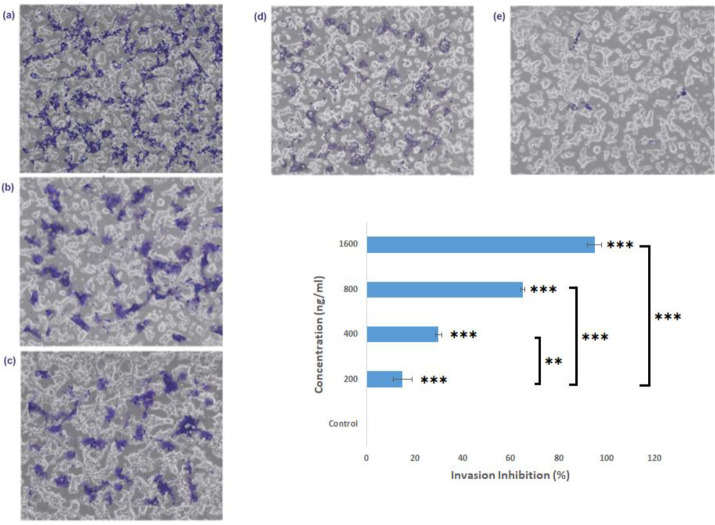
Effect of NB conjugated copolymer in preventing invasion of KDR cells (b-e) in concentrations of 200, 400, 800, 1600 ng/ml, (a) Control and (f) Anti-invasive effect on KDR cells (*** = *P*<0.001, ** = *P*<0.01), EC50 = 58.5 nM

Figure 13b–e shows the results of the invasion assay of NB conjugated copolymer at concentrations ranging from 200–1600 ng/ml. As indicated in Figures 13b to 13e, concerning the control group (13a), the NB conjugated copolymer prevents the invasion of KDR cells in collagen-coated transwells. This anti-invasive assay also has been shown in a graphical plot (*P*<0.001, EC_50_ = 58.5 nM. Similar to the migration assay, concentration-dependent cell invasion inhibition was observed for the H40-PEG-MTX-NB nanoplatform. The graphical plot indicated that the inhibition rate of KDR cells increases with increasing H40-PEG-MTX-NB nanoplatform concentration. Briefly, the highest percent of cell invasion inhibition was detected after KDR cells were treated with H40-PEG-MTX-NB nanoplatform at a concentration of 1600 ng/ml.

## Discussion

With the presence of properties such as good stability and solubility, lower manufacturing costs, antigen specificity, high affinity for the antigen, and less Corona effect in relation to antibodies, nanobodies have been evaluated as promising substituted therapeutic agents for cancer and anti-angiogenesis therapy. VEGF-specific monoclonal antibody/nanobody is the first anti-angiogenic agent that was approved by Food and Drug Administration (FDA) in 2004 and its effectiveness has been proven in several metastatic cancers (45). In this study angiogenesis inhibition of the H40-PEG system with an anti-VEGFR2 targeting agent and targeted release of MTX has been evaluated. This drug delivery system was carefully selected investigating the different formats of NB-based drug delivery systems such as NBs with intrinsic therapeutic activities (20, 21), nanobody conjugated with toxins (22), liposomes (23), polymer nanoparticles (24, 25), and conductor nanobody with carrier (26). Among them, nanobody conjugation and polymer nanoparticles are some of the widely used systems for their versatility in conjugating specific ligands for various proposes like co-delivery of drugs, using various targeting moieties, and engineering the structure and expected features in humans.

Effective targeting and angiogenesis inhibition properties of our H40-PEG system depend on surface modification, size distribution, the stability of NB, drug release profile, biocompatibility, and anti-angiogenesis properties.

Figure 5b shows that the prepared nanocarrier did not show any significant increase in size, which confirms its stability. In Figure 6, the SDS-PAGE analysis of the H40-PEG-MTX-NB after 2 and 4 months of conjugation has been shown. This experiment confirms the stability of our nanocarrier in terms of NB conjugation. Based on these findings it can be concluded that H40-PEG-MTX-NB is characterized by desired size, high drug loading capacity, and excellent colloidal stability for efficient cancer treatment.

The difference is in favor of drug release in acidic conditions (pH 5.5), as the release of MTX is accelerated in the acidic microenvironment (*P*-value<0.01). In other words, H40-PEG-MTX-NB exhibited a pH-dependent drug release profile and this may lead to increased intracellular trafficking of these nanoplatforms within tumor cells. 

Hemolysis assay was considered a rapid technique to evaluate hemocompatibility. It was found that fabricated nanocarriers were practically safe for blood components, in which even at a concentration of 1000 μg/ml the hemolysis level did not exceed 3%. This slight hemolysis indicates the hemocompatibility of the H40-PEG nanocarrier for the safe delivery of therapeutics within blood circulation. These results are well supported by WAN *et al.*’s findings, in which they developed antiEGFR antibody conjugated silica nanoparticles and the hemolysis level of this nanoparticle was around 3% (46).

Figure 10 shows that the toxicity of H40-PEG-MTX and H40-PEG-MTX-NB increased at high concentrations. H40-PEG-MTX-NB exhibited concentration-dependent cytotoxicity while the cytotoxicity rate of H40-PEG was negligible against cancer cells. As we expected because of the nanobody mechanism as a VEGFR-2 blocker, there were no significant differences between H40-PEG-MTX, and H40-PEG-MTX-NB in either MCF7 or KBR cell lines (*P*-value>0.1). So, intrinsically NB has no cytotoxicity properties and there is no difference in their cell culture assay. So, we used immigration and invasion assays in order to investigate H40-PEG-MTX-NB efficacy.

The results of the immigration assay have several similarities with findings of other researchers who fabricated HER2 nanobody decorated liposomes for co-delivery of nitroxoline and cisplatin against breast cancer. It was found that fabricated nanobody-conjugated liposomes containing nitroxoline and cisplatin could potentially inhibit MDA-MB-231 cells (an invasive breast cancer cell line) and confirmed the anti-angiogenesis properties of developed nanoplatforms (23). In invasion assays the findings are consistent with previous results as well, e.g., Zhang *et al*. developed HER-2-nanobody (NB)-conjugated human serum albumin (HSA) containing catalase (CAT) and chlorin (Ce6) for ovarian cancer treatment. Results unveiled that prepared nanoplatforms could efficiently inhibit tumor progression (47). The graphical plots illustrate the proportionality of the increase in the concentration of the H40-PEG-MTX-NB and the magnitude of the inhibitory effect of KDR cells.

PEG: Poly ethylene Glycol

PEG: Poly ethylene Glycol

NB: Nanobody

NB: Nanobody

## Conclusion

Anti-VEGFR2 NB-conjugated H40-PEG carrier loaded with MTX was properly fabricated and showed a promising outlook for consideration as a triple approach for anti-angiogenesis therapy. The use of nanobodies could provide a stable and efficient targeting approach for the nanocarrier. PEGylation of the nanobody could increase the half-time of the nanobody and mask any possible toxicity associated with the nanocarrier. Moreover, the hyperbranched properties of Boltorn H40 could significantly deliver more therapeutic agents to the targeted cells. In this work, we briefly show the synthesis route and some of the *in vitro* analyses of the developed nanocarrier. H40-PEG-MTX-NB nanoplatform with a particle size of 105 nm displayed a pH-dependent drug release profile in which the release of MTX was accelerated in the acidic condition. This unique feature is highly desired for targeted drug delivery and efficient cancer therapy. *In vitro* biocompatibility assays revealed the safety of fabricated nanocarrier. It is expected that the developed H40-PEG-MTX-NB nanoplatform could effectively inhibit tumor vascularization and surprisingly the obtained results from cell migration and invasion assays have further strengthened our confidence in the anti-angiogenesis properties of fabricated nanoplatform.

In this work, we did not prepare BALB/c mice with KDR over-expressed receptors, so the *in vivo* analysis of our anti-angiogenesis carrier could not be accessed. Hence, in future, this assay can be addressed and the result will be depicted in our next study.

## Authors’ Contributions

SMA Contributed to data curation, investigation, resources, visualization, and writing the original draft. HR Contributed to data curation, writing, review, and editing. MB and SJ TR helped with supervision and resources. AR Contributed to conceptualization, funding acquisition, resources, supervision, writing, review, and editing.

## Conflicts of interest

The authors declare that they have no conflicts of interest.
